# Reasons for illicit drug use in people with schizophrenia: Qualitative study

**DOI:** 10.1186/1471-244X-10-94

**Published:** 2010-11-22

**Authors:** Carolyn J Asher, Linda Gask

**Affiliations:** 1 Pennine Care NHS Foundation Trust, Stepping Hill Hospital, Stockport, UK; 2 School of Community Based Medicine, University of Manchester, NPCRDC, 5th Floor, Williamson Building, University of Manchester, Oxford Road, Manchester UK

## Abstract

**Background:**

Drug misuse is an important clinical problem associated with a poorer outcome in patients who have a diagnosis of schizophrenia. Qualitative studies have rarely been used to elicit reasons for drug use in psychosis, but not in schizophrenia.

**Methods:**

Seventeen people with a diagnosis of schizophrenia and who had used street drugs were interviewed and asked to describe, in narrative form, their street drug use from their early experiences to the present day. Grounded theory was used to analyse the transcripts.

**Results:**

We identified five reasons for continuing street drug use. The reasons were: as an 'identity defining vocation', 'to belong to a peer group', due to 'hopelessness', because of 'beliefs about symptoms and how street drugs influence them' and viewing drugs as 'equivalent to taking psychotropic medication'. Street drugs were often used to reduce anxiety aroused by voice hearing. Some participants reported street drugs to focus their attention more on persecutory voices in the hope of outwitting their perceived persecutors.

**Conclusions:**

It would be clinically useful to examine for the presence of the five factors in patients who have a diagnosis of schizophrenia and use street drugs, as this is likely to help the clinician to tailor management of substance misuse to the individual patient's beliefs.

## Background

Illicit drug use is common in schizophrenia. Reported prevalence rates vary, for instance, in a recent study 11.9% of people with schizophrenia had comorbid drug abuse or dependence [1]. A recent meta-analysis showed about 1 in 4 patients with schizophrenia had cannabis use disorder [2]. This is up to five times higher than in the general population [3] and results in higher rates of relapse, hospitalisation, suicide and other adverse outcomes [4]. The reasons for this comorbidity are complex and a number of competing theories have been generated and studied using quantitative methods [5-8]. Reviewers have sought to evaluate the degree of empirical support that exists for each theory [6,9]. Psychosocial factors appear to be important in maintaining substance use in this population [5,6,8,9] and a thorough assessment of psychosocial factors is important in engagement and tailoring interventions [5,6,10]. To answer the question as to why this client group uses substances, it makes sense to discuss this directly with service users [4,11]. From the quantitative literature, self reported factors which may account for drug misuse in schizophrenia have been summarised: to achieve intoxication, to enhance ability to socialise with others, to self-medicate for positive and negative symptoms of schizophrenia and to relieve dysphoric mood; in the case of cannabis but probably not other substances, the cannabis use itself may have precipitated the schizophrenia in vulnerable individuals [6]. Quantitative self report studies have been very useful but may fail to discover some important reasons for drug use in schizophrenia because the questions posed are fixed in advance of any data collection. By contrast, a number of qualitative methods involve constantly analysing the data as it is collected and adjusting the questions posed so that the researcher can refine the questions to test out new concepts in subsequent interviews [12-14]. Novel reasons for phenomena, uncovered using qualitative methods, can later be tested in larger groups using quantitative methods.

To this end, two qualitative studies in the United Kingdom (UK) have recently looked at reasons for drug use in patients with psychosis [15,16] and a study in the United States of America (USA) has looked at attitudes to substance use in a mixed group of patients, some of whom used drugs [17]. A further UK study of reasons for drugs and alcohol use in people with schizophrenia used mixed methods including what appears to have been a very small semi-structured interview study and a descriptive analysis of tapes of therapy sessions (the method was not well described) to develop questions which were then posed to a larger group and factor analysed [18]. These studies have found that reasons for drug use were: to relax and improve social performance [15,18]; to belong and share in a group experience [15]; to avoid losing a peer group [15]; to achieve intoxication [15,16]; to reduce side effects of medication [16]; to reduce aggression [15,16]; to cope with distressing emotions and positive symptoms [18]; to feel powerful/creative [16,18]; to cope with trauma or loss [15,16]; to achieve a sense of identity and social status, escaping a dull life [15,16]; because drugs were not believed to cause psychosis [16]; because the preferred substance was more acceptable in the hierarchy of acceptability of drugs [15]; because cannabis had been used long before onset of psychosis and was normal in their community [15,16]; because cannabis was like a medicine [16]. Examples were found both of patients who thought that drug use had been a factor in precipitating and relapsing mental illness and patients who denied any adverse impact on mental health [15-17]. Reasons for attempting to not use drugs were because of negative effects on mental state [16]; cost and illegality [16]; to improve health, finances and family relationships [15].

It remains unclear however whether the results of qualitative studies of reasons for drug use in psychosis would be applicable to the narrower sub-group of people with schizophrenia; thus our study looks specifically at reasons for drug use in schizophrenia.

The aim of this study [19] was to elicit reasons why some people who have a diagnosis of schizophrenia repeatedly use any street drugs, using a qualitative methodology so that novel reasons could emerge and existing concepts might be examined in the light of participants' experiences.

## Methods

### Design of Study

Qualitative study carried out with people with a clinical diagnosis of schizophrenia. Ethical approval was obtained from Bolton Local Research Ethics Committee (LREC) and subsequently from Central Manchester LREC, reference numbers 02/BN/704.

### Participants

Participants were people from two socially deprived areas of Greater Manchester, an inner city area and a smaller town within the conurbation. All had a diagnosis of schizophrenia, used substances and were known to local psychiatric services. Participants were not under the clinical care of either of the researchers. Participants of diverse demographic (age, sex, ethnicity) characteristics were sought in order to obtain a maximum variation sample [13]. We approached all consultant psychiatrists in these services asking them to identify all service users who met our inclusion criteria. Recruitment and initial contact with the patients was by an opt-in letter sent on behalf of and with the agreement of their own consultant. We sought to recruit all those who met our inclusion criteria and were female or of Black Minority Ethnic (BME) groups; we recruited as many white male participants as were necessary to reach saturation of data (see below). To compensate for the difficulty we encountered in recruiting female clients and people from ethnic minorities, such patients were purposively sought by identifying potential interviewees from these groups and repeatedly requesting consultants to pass on opt-in letters to these patients in particular.

### Interviews

We asked individuals to describe in narrative form their history of drug misuse and mental health problems from earliest experiences, moving forward in time to the present, with concurrent descriptions of their social context.

We wrote an initial topic guide based on the literature as follows:

◦ "What substances have you ever used?

◦ Tell me about when you first started using substances.

▪ What was life like at the time?

▪ What effects do you get from each substance?

◦ Tell me about how your substance use has been over time since then.

▪ What has life been like?

◦ How have you been in yourself?

▪ Does anything help with that?

◦ What are your opinions of different street drugs?

◦ Why do you think that people who have psychosis would carry on using substances?"

The interview covered items in the 'topic guide' and any additional material spontaneously suggested by the patient. We adapted the order and style of questions at each interview in response to cues from the participant. To gain the maximum information, all participants were encouraged to give their own detailed personal account of their drug use history in a chronological manner, with minimal prompts from the interviewer, including any associated memories or ideas that were meaningful for the participant. The interviews were for as long as it took for the participant to tell their story or as long as the participant could tolerate, hence they ranged from approximately 40 minutes to 2 1/2 hours. They were provided with snacks and could take breaks if desired.

### Analysis

All interviews were recorded, transcribed and anonymised. The transcripts were analysed utilising Grounded Theory [14]. We read each transcript and added meaningful labels or 'codes' against words or phrases that were relevant to possible reasons for illicit drugs use. We constantly compared codes within and between interviews and condensed similar codes together. We analysed the data whilst we continued to carry out more interviews, adapting our topic guide as the study progressed. At all stages of the analysis, we compared our emerging ideas about reasons for drug use with the interview transcripts and we discarded any ideas if the data did not support them. We wrote lists of codes for each participant ('open coding memos') initially grouping the codes according to descriptive headings of which substances were used, how they were used, any unusual incidents, the individual's life/relationships and perception of self. We compared these lists between participants to look for meaningful groupings of codes or 'categories' and wrote 'theoretical memos' about possible causal links between categories. Our theoretical memos included inductively writing a 'story line' or composite of the interviewees' stories of their street drug use and constructing a wall chart of the data to look for emergent patterns [20]. Wherever we found that the 17 participants could be divided into two or more groups according to a characteristic relevant to drug use, we closely examined how the groups compared and contrasted to explore why these differences occurred.

We continued recruiting subjects and analysing interviews until we had reached saturation of the data, in that there were no new themes emerging and we had tested all the categories for disconfirming cases and variations.

## Results

Forty-five people were sent opt-in letters, of which 27 agreed to receive further information. Of this 27, 17 participated (see Table 1), one did not supply contact details, one was unable to consent due to acute psychotic illness, three declined without giving a reason and five declined, stating that they felt unwell.

**Table 1:** Characteristics of participants: N = 17

**Gender**			
Male	16	Female	1
**Age**			
16-19	1	30-34	1
20-24	4	35-39	10
25-29	0	>40	1
**Ethnicity**			
White	13	African	2
African-Caribbean	1	Asian	1
**Current illicit drug use**			
Using	12	Abstaining	5
**Street drugs used**			
Only cannabis	3	Multiple but mainly cannabis	6
Mainly stimulants	6	Mainly opiates and stimulants	2

To compensate for the difficulty in recruiting female clients and people from ethnic minorities, such patients were purposively sought by identifying potential interviewees from these groups and repeatedly requesting consultants to pass on opt-in letters to these patients in particular. We specifically sought these groups (with some success - see table 1) in order to get as near as possible a maximum variation sample and hence make our findings more generalisable.

In reviewing our 'theoretical memos', the most fruitful comparisons appeared to be between those who intended to abstain in the future and those who preferred to continue street drug use.

We identified five key reasons for street drug use in schizophrenia. Drugs were used:

• As a identity-defining vocation

• To belong to a peer group

• Due to feelings of hopelessness

• Due to beliefs about symptoms and how street drugs influence them

• As an equivalent to taking psychotropic medication

### Drug use as an identity-defining vocation

Like a vocation, the activity of substance use was often acquired in youth and developed with increasing knowledge and skill over time, providing a sense of identity, a social activity and enhanced self-esteem through mastery of a subject.

Almost all participants first tried illicit substances in their teens and fifteen had commenced drug use before developing mental health problems. Just as hobbies are often thought of as 'keeping young people out of trouble', some believed that cannabis use was protective against use of drugs such as heroin or indeed against use of excessive alcohol.

A white male participant, in his late 30 s who had injected amphetamines and used LSD and cannabis heavily, gave up drugs for a partner and became alcohol dependent; he said:

"They didn't realise I was taking overdoses and things like that. Because every time I took an overdose, it was paracetamol, 100 at a time. Where's the life gone. "I'm not a piss head [alcoholic] what have I done? Dad's a piss head, mum's a piss head. I'm a junkie [drug addict], where's my drugs gone?" All the drugs gone out of my life. I was f***ed up in my head because I was on a different way of life." (Participant 1).

He went on to describe drug related aspects of his identity that he felt were positive and that he had decided he could retain despite abstinence by convincing himself that the drugs literally remained inside him long term like an *"everlasting gobstopper*" [fictional children's confectionery]. When the interviewer asked him why, he said:

"Because [pause] when I was a teenager, people out there, the society, people popping e's [ecstasy], having a bit of China [heroin]. I tell them to their face, I say, "f*** you're head up, I've done it before I don't want it." "You keep taking that", I was telling someone, I'm a big grass[sneak], f***them. "Listen to me what I'm saying". Like on bus-stop, on way home from Manchester this afternoon, couple of lads. I'm there, the famous laddy [boy]. The "mad junkie". I got a lot of friends. I was at the bus stop and there was a lad [boy] there talking about something. I knew what he was on about, he wanted to know about this that and the other. Said, "how do you do this and how do you do that?", these things, drugs. "What's best to take?" I said, "don't take any more". A lad next to me, I said "look am I happy? I'm f***ing straight [off drugs], I'm not a 'mad' you know, just 'f***ing mad'." To prove to him, I said, "don't bother taking drugs and tell all your mates in [suburb] don't bother taking them either". But he said, "I want to do it". But he who laughs last laughs last. Me and my friend [name], before he left me,, we was injecting speed. We would do a lot of injecting, a lot. Before he died. About 6 or 7 years back. We left off, we was in a night club in Preston, the [name], you been there? It's hardcore [good]. We left each other. We're twins, best mates, always together, solid to the world." (Participant 1).

When the interviewer asked if he meant they were encouraging each other to take more drugs, he replied:

*"We were like fanatics, like professional whizz heads [users of amphetamine]. Professional whizz heads. We did it, we did it. Never stopped for a second of the day. We'd sleep for 4 days. In bed for 4 days. Sleep 4 days gone, no bullshit [lie]. Valium [diazepam] 15, 20 mg, temazepam as well. Bed for 4 days. I'd not seen him, about 8 month after, he started dying of angina of heart attack and died. He, he died. Swine he was." *(Participant 1).

Most felt that they had a lot of knowledge and experience of drugs. As expressed in the quote above, drug use was an important part of their identity.

The majority had started with cannabis and then tried other drugs. A British Asian Muslim man in his thirties who mainly used amphetamine (by mouth) explained:

*"I've tried whole range of them really since I was a teenager. started off with cannabis to begin with then it moved higher and higher to, acid tab, ecstasy, blue and all of that, e tablets come out and on them. Amphetamines as well. So carried on with the whole range of them, but I didn't like the cannabis I didn't like cannabis. I preferred the uppers [stimulants] rather than downers [depressants], but started taking some of the uppers. It was really one at a time. I've quit it all now, it got too much for me over the years, amphetamines." *(Participant 16).

Cannabis use was often seen as 'normal' among older people that they looked up to when they were teenagers, including elder brothers and sometimes parents. The same participant explained how he had first experimented with what he thought was his father's cannabis:

*"Well someone in my school, a boy [name] he's the one who found some. My dad used to smoke it and I found a piece of my dad's but it wasn't real at the time, a real piece, but he asked me to make him some joints [cannabis cigarettes] out of that." *(Participant 16).

Similarly, a man in his early 20 s of African descent, who used mainly cannabis and alcohol, but also opiates, LSD, cocaine, amphetamines and benzodiazepines said:

*"One time [my mum] had to be admitted into hospital, so for three weeks my brother was looking after us in the house. So we had all these friends in and, I remember my brother was really protective of us then and he had his friends smoking buckets [cannabis apparatus], smoking cannabis in the house. And he wouldn't let me go near it. But on other instances they had a couple of joints [cannabis cigarettes] and they used to save me some cos I was [his] little brother. look after me that way." *(Participant 3).

For sixteen interviewees, substance use had been the main leisure activity or an essential part of their life for much of their adult lives, although four of these had stopped using substances at the time of the interview. Four described having a kind of 'connoisseurship' of substance(s), in the sense of having in-depth knowledge of the varieties of a substance and technical aspects of these. A white male in his late 30 s, who regularly uses cannabis, including to relieve anxiety and to feel more musical and who had not lived up to parental academic expectations, described how to make a cannabis cigarette:

*" If you heat it, it expands, but in a lot of places in the world, they'll frown upon you for heating it because it burns off the top notes, um so with 'Squidgy Black'[a type of cannabis] you could just roll it into a sausage and drop it in and that was pretty incredible." *(Participant 4).

All but three interviewees clearly described a hierarchy of acceptability of substances, including one patient who had been dependent upon heroin. In this hierarchy, cannabis was seen as acceptable, whilst crack cocaine and heroin were least acceptable. Cannabis use was sometimes seen as protective against use of other substances. Another white male participant in his late 30 s who had used alcohol, solvents, pills, poppers and glue, and had tried but disapproved of heroin, amphetamine and cocaine, said that it was helpful to decide that he preferred cannabis:

*"...It's better, if you are on [using] something, because you are not tempted to be on what they are on. If you are with your friends and you state your case that you don't touch that*..." (Participant 6).

### To belong to a peer group

Substance use also offered a sense of belonging, which appeared from the data to be both highly important for the individual but also conditional upon continued substance use and greater efforts to fit in. For almost all the interviewees, (15 out of 17) beginning to use substances was like a rite of passage, as if to mark the joining of a community. Participant 6 described above, who said he preferred cannabis, described vividly the sense of togetherness enjoyed through substance use:

*"Sometimes when everyone's that tied up, this is my experiences, everybody can sit in a room and there's drugs on that table, right so we all take the drugs that we decided. Now he's worried he might o.d. [overdose], pop his clogs, [laughs] he's worried that he might o.d. Now all the time we're comforting each other, talking to each other, on this drug, talking people round 'because we've not been given it off the doctor, it's come off the street. And all the time even though we're laughing and enjoying a joke, each one is holding each other up all the time, looking out for [protecting] each other, it's just natural. Really strong men and their weaknesses, because it makes them feel weak, they don't know if it's going to pop them off, so then they're all comforting each other and eventually it gets to a point where everybody is okay and everybody will start breaking off, wandering up there or coming back, that's what's so good about it"*. (Participant 6).

Participants said they had been urged to use drugs by friends or, more usually, that patients sought substance-using peers. However all had *persistent *difficulties with social interaction. Reasons included being distracted by hearing voices or experiences of their thoughts being interfered with, having lack of drive to socialise, anxiety or low/irritable mood, feeling stigmatised and being preoccupied with unusual interests or experiences. Eleven out of 17 interviewees described how drugs helped them to mix and talk to others. Some said drugs only helped them to mix with people who *also *used drugs. Indeed sometimes drugs made it *harder *for people to mix with people who *didn't *use drugs. A white male in his mid thirties who said he was given amphetamines age 16 by his elder brother and who continued to use with this relative said:

*"No I don't usually see anyone or hang about [associate] with anyone who doesn't take them, I don't like people's attitudes, you know I'm soft me I'm very kind at heart so I only like hanging about with people who understand me." *(Participant 13).

However giving up drugs would mean, for some, having to lose their friends and twelve people reported that they felt they had to continue to use drugs in order to keep their groups of friends. For example Participant 6 who now preferred cannabis, explained that he needed to use cannabis when with peers and would come under pressure to experiment with 'pills':

*"...If you are with your friends and you state your case that you don't touch that but you want to be friends with them, then my mate used to come back and say that they had sorted you out [bought you] some tablets for tonight, you can have a laugh [good time]. 'Becauseit's no good being with everybody I knew, because you just can't blend in at all, you just can't have a laugh because, they're on a different level." *(Participant 6).

Many said that they had been taken advantage of. This included getting into debt with drug dealers and giving drugs away. Self esteem was experienced as higher in the context of the subculture of substance-use as compared with in mainstream society. Participants could be seen as one of the gang, heroes who had bravely saved others from danger, wise elders, connoisseurs, admired risk takers, intrepid explorers of the mind, entrepreneurs or generous sharers.

There was strong evidence of people hiding their hearing voices from their substance-using peers for fear of being labelled as ill, but it appeared that such peers were more tolerant of the types of unusual experiences as might be explained away as being due to substance use. In contrast, some reported that if they did begin to have experiences beyond what their peers judged to be typical, they would be informed in a helpful way. A white male in his thirties who regularly used intravenous amphetamines, sometimes used cannabis and had tried heroin, explained that after his first episode of schizophrenia, his old friends had abandoned him, whereas people who use drugs "*care about one another their well being*", they had visited him in hospital, they enquired how he was and he believed most of them had experienced "*paranoia*".

*"...They can handle it [pause] when I've been paranoid, when I've been on drugs, I've been paranoid, they say like, 'stop taking drugs, you're paranoid, you're ill'"*. (Participant 5).

This individual blamed his substance use on the mental health services for 'introducing' him to people who use substances and assuming that he did too.

### Feelings of hopelessness

Areas of their lives about which some felt hopeless included relationships with partners, family and friends, acceptance by the wider community, employment prospects and accommodation. Where participants were optimistic about improvements in these aspects of their life, and if they saw substance use as a potential barrier to something that was otherwise attainable and strongly desired, then they spoke of being prepared to give up substances.

A white male in his late thirties who used heroin, crack cocaine, amphetamine and cannabis, said he had decided to abstain from opiates and stimulants in the hope that he might gain employment and resume contact with his daughter:

*"...But when she's older she's going to have to look at me as a father figure and then I'm going to have to have qualifications behind me so I can show hersomething, so a mechanics course or somethinglike that. And welding courses, so I can communicate properly with her so she can look and say 'oh my dad's a mechanic' or 'my dad's a computer controller'. Not just a drop out." *(Participant 8).

Loss of loved ones was commonly mentioned. Four described at least one significant bereavement, and six reported having experienced a prolonged rejection by their relatives at some point in their lives. Twelve reported losing friends or girlfriends, due to rejection in the context of developing symptoms or continued substance use or more rarely because of deaths due to substance use. Most participants said they felt somewhat outside of society. A white male in his twenties who preferred cannabis but when younger had used a wide range of other substances had a girlfriend but was unable to retain work due to persecutory voices:

*"I don't see my family very often, I don't have any friends, there is no real good things*" (Participant 7).

Participant 6 (described earlier) who had had a difficult middle phase of his life in which he had switched from drugs to alcohol and felt he had to 'get violent' to access mental health services, but was an inpatient at the time of interview, described use of cannabis to reminisce about lost relationships:

*"I use it more as a comforter now as I've got older, but more for just mucking around [recreation]. What makes people keep using substances? That's one of the main things I can think of. The other thing is, with me it takes me back to my childhood. Some people might get sores done in [injured] by society and they need something that'll shut the body down for a while. So they might get their head back together because they feel so horrible or feel so poorly or they're being victimised by society or something. They take it just to, [have a] quiet life. It's just like going on holiday, a cheap holiday! Who gives them a break, then hopefully you'll wake up in morning and you'd be ready to take on the world" *(Participant 6).

Many had thought about stopping drugs so that their lifestyle would be more stable. An Asian male participant in his thirties described how in the past he did not fit in with cultural expectations including of working, but that he believed that his abstinence from stimulants could result in his finding employment and getting married:

*", , , , , I'd like to get married at the end of the year and then move." *(Participant 16)

He went on to say that his main incentive to remain abstinent was that it would enable him to buy his own house, (his family home is difficult due to mental illness in elders and drug dealing siblings). However the initial stimulus to abstain had been concerns for his physical health:

*"Well it got too much for me really, I'm getting older and, if I don't let go of it now, then it's going to be in my system and it's going to get a bit too much tricky on the heart*." He continued, "*All the power in you, so that amphetamine makes your heart pound faster. So you're not supposed to have anything make your heart pound faster when you're older"*. (Participant 16).

Others preferred to continue using substances and did not wish to seek regular employment, although some continued using drugs because they believed drugs helped them to carry out certain tasks, such as artwork, music, study, muscle building exercises and household chores. Some aspired to goals that seemed difficult to achieve in the hoped-for timescale. Examples included working in USA, being able to afford to driving lessons and a car on limited savings, or of starting in highly skilled jobs. It was difficult to establish in the interviews why they had settled upon such goals, as any challenge to these seemed to threaten rapport, but there was some suggestion that they felt entitled to a better standard of living but had limited experience of working steadily towards realistic goals. There was some evidence that having unrealistic plans might lead to a cycle of abstaining in the hope of some reward, but when that was not achieved, being very disappointed and rapidly resuming substance abuse to cope with the feelings. In two participants' interviews there was an example given of using drugs when high hopes or expectations were disappointed. There was use of substances simultaneous with experiencing disappointment in a further eight.

Explaining about his recent frustrations with not yet being provided with independent accommodation to move on to, Participant 1 described above (white male, late 30's) said:

*"They're not doing anything for my life I've got to do something about it now. Try a bit of whizz [amphetamine]. See what happens"*. (Participant 1).

Being in very poor accommodation occurred at some stage in the lives of twelve out of the seventeen interviewees. Some participants reported that being in a hostel had resulted in being a victim of crime or other adversity and using drugs to cope. Eight participants reported an episode of problematic accommodation, such as a hostel, during which they had escalated their substance use, in terms of quantity and types of substances used.

### Beliefs about symptoms and how street drugs influence them

Those who believed that they were not psychotic and that street drugs did not usually have a deleterious effect on their mental state were less likely to be amenable to abstaining. 13 out of 17 participants currently regarded much of their voice hearing and other unusual experiences as real. Such experiences were often of a religious or persecutory nature. A white female in her late 30 s who used mainly cannabis (to control anger) and amphetamine (to cope with unusual experiences), explained about her use of amphetamine:

*"It helps me fight my abusers off and if my abusers get too heavy; I've been having illegal operations and all sorts happening to me. Now these operations are not ordered by medics at this hospital or even my doctor at this hospital. There has been an illegal operation done on me only a few days ago while I was pregnant which could be due to the fact that I could miscarry. These operations are due to a childhood abuser of mine getting in to the surgical realm, studying surgery as he got older and operating on me, he's been operating on me since I was about 18,19 and he's done some nasty operations on me, but he is no longer a problem." *(Participant 12).

Sometimes medical labels were used to describe distress, but in most cases, interviewees' meanings of such term were very different to the DSM IV definition. For instance, Participant 6 defined 'psychosis' as "*a feeling of paranoia, um feeling like the world's racing by faster*". Participant 7 described above, who intended to continue use of drugs, believed that incidents of (ordinary) curiosity about sexuality as a child had resulted in "*schizophrenia*", by which he seemed to mean anxiety due to 'real' persecution by others who had misunderstood his behaviour:

*"Because I've grew up with schizophrenia, well I've grew up with people thinking I'm some sort of sexual menace, that's my degree of schizophrenia, I've grew up with people thinking I'm some sort of sexual menace, when really I'm not, if anybody really knew that they would know I'm the sweetest guy and I would never hurt anybody, and I really mean that, and I don't mean, I'm sure there are some paedophiles out there that think well to touch somebody up a little bit doesn't really hurt them, it does,, and I would not lay my hands on anybody and touch them up, but there's just so many people questioning me, I question myself" *(Participant 7).

Twelve did not believe that substances had a consistently negative impact on the severity of voices or preoccupation with unusual beliefs. Such views were mainly based on experience and sometimes because voices were believed to be real and external to self. A white male in his thirties who was currently using cannabis (but had tried gas and solvents), explained that he had initially blamed his voices on cannabis, but had subsequently experienced a worsening of the voices whilst abstaining and so had decided to resume use to cope with his anxiety.

*"...after about a couple of weeks the voices got steadily and constantly worse, even though I wasn't using drugs whatsoever and I thought to myself, well I was relieved a little bit when I was on the weed so I went back on it and I just relaxed then and made me able to cope with the voices a bit better" *(Participant 14)

Two stopped using cannabis after they began to hear voices; of the remaining participants, nine had mainly enjoyable/grandiose voices and six had voices that were distressing but modifiable with substances. Four had unpleasant voices but chose to use substances to attend *more *to these voices rather than try to blot them out. In the following two examples, amphetamine was used for this purpose. A white male in his thirties who was living in supported accommodation and used multiple substances including opiates, stimulants and cannabis described the effect he hoped for from amphetamines:

*"I just get chatty to the voices talk to them, talk about processes and about the book I'm writing, you know, about the science fiction book I'm writing and the processes what I've been taught, through hypnosis. You know, that's what they're after, see and I won't give them them." *He went on to explain that it was risky to be 'chatty' with the voices. *"I go again, 'you talk a load of crap, as far as I'm concerned, you never tell me anything' and they're always trying to control me. And I was trying to find out about the 'special forces' implants what they put in my head when they make me safe. Which means so I can't be hypnotised [deep breath] you know. [Cough] But I can't say much else, you know because I think they're listening in to our conversation*" (Participant 8).

Participant 12, described above (late 30 s female), said:

*"I do take amphetamines every now and again, now amphetamines I do use on the odd occasion when I'm having to stay awake because of expecting influxations [the arrival] of abusers." She continued. "So it's a false energy burst basically and really I use that to manipulate my body in to staying awake so that I can deal with any abusers that might hurt me." *She continued. *"It involves me getting a bit rough with my abusers, but I've learnt a crafty way of doing it. At the moment in the psych ward, it's a very unusual psych ward that I'm on, it's actually got an electric roof and the abusers have actually been going in the roof and down through the ceilings and abusing people and I go on the roof and I collar [grab] them on the roof and I actually do use the electricity on them to stun them, so the police and army can arrest them." *(Participant 12).

Amphetamine use was repeatedly described as concomitant with unusual experiences, but was seen at the time as raising alertness to engage fully with the experiences, rather than the amphetamine causing hallucinations. A white male in his thirties who was now abstaining said:

*"I just wanted to be out of my head, it was like, with my psychosis, the more I was out of my head the more I was in touch with mental illness. the quicker my mind was my metabolism obviously speeds up my mind thinking quicker and this thing that was going on in my head I wasn't sure if it was real or make believe or just illness know. But I knew I had to be alert you know to get myself through it and I mean I've been there and I've been in hospital and I've actually visually and audibly with my hands created the universe just in my mind, but seeing it in front of me as if I was a god. I've created this universe, well a galaxy it was, spinning yes and things like that. And I thought that I had to have this amphetamine to keep me on that level"*. (Participant 17)

By contrast, five people said that cannabis could allow them to let the voices wash over them without causing distress, including this white male in his thirties:

*"I just treat it as um just sit back and relax and sort of go with the flow sometimes, I'll hear the voices and I'll go "yeah, yeah, yeah carry on, yeah, yeah, yeah carry on I don't care what you say."" *(Participant 14).

Although all agreed that some street drugs cause some increase in some unusual experiences or beliefs, ten participants intended to continue their use in future. Reasons given by these ten were that only a specific drug or bad batch was to blame for the experiences (seven), that only some experiences are caused by substances (nine) and that street drugs allowed better coping with these voices/beliefs (ten participants).

An example of an adverse incident with drugs being blamed on a contaminated sample and drug use continuing or even escalating thereafter came from a white male in his thirties who used amphetamine from age 17 (with his elder brother):

*"When I was 21 I used to have it but it wasn't very good stuff, then I got poisoned. I thought I was taking amphetamine but I don't know what it was, and it done something to me." *He explained how he knew this had happened. *"Because my muscles all felt weird, it did something to my muscles, spasmed them out. When I was 21 and it affected me for ten years that, it was only 2001 that it actually went away, I knew it would go eventually but I needed good amphetamine to get rid of it, that's what I discovered. Because it took all my strength away and amphetamine gave me strength so I was fighting against it all the time. My muscles felt like someone had hold of my arm all the time [he gestured as if being restrained]"*. When asked if he meant someone was not letting him go, he continued. * "Well no I could feel as if someone had, that's what it felt like, my muscles felt like someone had hold, there was something wrapped round my arm or someone touching me, a feeling all the time on my arms and leg muscles. But I just kept on persevering and kept on fighting it and kept walking and trying to get strong and trying to get strong and then I'd be coming down [withdrawing], I'd have to get more amphetamines the next day and going through ten years of doing that, and eventually I woke up one day after doing a detox in hospital and in prison and I realised "god it's gone it's gone", I couldn't believe it" *(Participant 13).

A man of African descent in his late forties who used cannabis but had also used amphetamine in the past explained:

*"The problem with marijuana is you know it is not the same all the time, it is rubbish most of the time that is one of the problems. If you could standardise you could decide, you could think better with it you see, it's changing all the time so it's difficult to think with it so [laughs] you know it's difficult to think with it"*. (Participant 11).

This meant that he kept using cannabis in the hope that the next batch would be a 'good' one.

Participant 15, a white man in his twenties who had used cannabis intermittently since age 15 and thought he would continue to do so, said that cannabis *"kills [brain cells] off"*, thus *"over the years it could make you lacking confidence"*, and he thought it made him *"paranoid*", meaning *"People out to get you name calling you behind your back and stuff. You just think they're doing it but maybe they're not"*. However, cannabis was his way of coping with voices and 'paranoia', in order to *"relax, just forget about things*". He was well aware of the contradiction and found this so stressful to discuss that he terminated the interview.

Four people reported that they had had *more *unusual experiences when they abstained from substances than when they were using them. For instance this white male in his thirties who started using cannabis age 25 (following a bereavement and resultant family breakdown) began experiencing 'pressure' from voices soon after:

"*From the voices, just laughing as if they was, I mean I was in a house where you couldn't see in, but they could, they was out there, "oh he's doing this and doing that and doing this and doing that" and then having a giggle about it and I just lost it [became mentally ill] and that was it then, I stopped smoking completely, weed [cannabis] and normal cigs [cigarettes]*." (Participant 14).

When asked if he thought there was a link he said. "*With the weed yeah, at first I did and then after about a couple of weeks the voices got steadily and constantly worse, even though I wasn't using drugs whatsoever" *(Participant 14).

Many denied any dose-response relationship between substance use and psychotic symptoms. Two considered that voices were reduced by substances. Participant 11, described above said that the effect of cannabis on voices was "*I think maybe keeps it quiet*", however he believed that the available cannabis "*lacks potency*", hence "*it keeps it quiet but not as quiet as I think it can, you know I don't know how quiet it can keep it but I think it can keep it pretty quiet*." When asked what he would have to do to cannabis to make the voices quieter, he replied:

"*You know if it if it has got the right potency because cannabis is like apples, some apples are not so good, some bananas are not so good, or for example aahh! See cannabis is like that, so we have to learn how to cultivate it, cannabis with ears that's black, standardise it like that" *(Participant 11).

It was important that the substance be taken in modest quantities to gain optimum effect:

*"If I take about half a gram, or two half a gram a day, it give me a feel good factor, I feel right again, I don't feel paranoid or anything. I've tried taking more than that, but then it gets to me." *(Participant 5).

If too much of the substance was used, negative effects could be experienced. Participant 4 (described above), who regularly used cannabis, had been detained in a psychiatric unit and said he had "*escaped a few days ago and had one joint*" because he was craving "*just desperately wanted some*". Cannabis "*shouldn't be done every day really*" but "*it can get that way though*", in which case he can experience "*short term memory failure*" and "*that's what you have to be careful of*". He said that using cannabis once per three days was ideal for him but "*that would be very hard to stick to*" because "*it's hard to get control over it*" due to its being "*psychologically addictive*". (Participant 4). Like other interviewees, he appeared to see substances as inherently challenging, like mountain climbing, thus they could not be fully mastered.

Many who did not regard hearing voices or other unusual experiences as illness, did regard themselves as having problems with mood or anxiety. Almost all described using substances to treat mood, sleep, appetite, or anxiety problems:

*"It seemed like everybody knew that I were blessed and everybody just wanted to pull me down, so that's when I started using drugs again." *(Participant 6).

Some also reported having improved functioning on a limited dose of substances. A white male participant in his twenties who used cannabis described the effects "*like a slight dose of hyperactivity*", he clarified "*cannabis makes you feel better*"; and "*lifts you mood as well*" and *"makes you more confident and makes you want a conversation more" *(Participant 15). This phenomenon of using a small amount of a substance to enable them to carry out particular tasks has also been described earlier in the 'hopelessness' theme.

### Viewing illicit drug use as equivalent to taking psychotropic medication

Many participants commented that prescribed medications were in many ways equivalent to illicit substances:

"...[cannabis is] *a bit like when they give you medication, then it sometimes takes two week to kick in [take effect]*". (Participant 6)

Participant 12 (described above) explained how she used cannabis to avoid getting aggressive on the ward:

*"Haloperidol takes about half an hour to work, now if you need an emergency sedation, if you're going to do any damage, you're going to do it before the sedation works...Yeah, cannabis works within a few minutes." *(Participant 12).

This meant that street drugs were useful instead of or as an adjunct to prescribed medication. Ten thought that health professionals were unfair or hypocritical for saying that patients shouldn't use substances, but *should *use medication.

Participant 6 believed he would "*get better*" if he used antipsychotic medication and cannabis in combination (although he would avoid mixing alcohol and medication): *"You're better off just having a couple of joints [cannabis cigarettes] and getting better that way"*. He also explained how he had used cannabis as an inpatient:

*"While I were in here cos I was slavering [dribbling saliva], and just kept getting the slobbers [dribbles] all the time, all over my top, it was horrible. So I started smoking cannabis because cannabis gives you dry mouth [laughs]. It worked too well, but they weren't too pleased, they took me off the cannabis and gave me tablets instead, they weren't too pleased I'd used it. But it stopped my slavering*." (Participant 6)

He believed that nurses didn't want him to use cannabis because they didn't like the fact that he had "*solved my problem of the dry mouth*" himself independently of their control and because:

*"What they were looking for was to see what the clozapine were doing for me they didn't want, if I take cannabis it would have blocked it out you see and they couldn't have their study properly. Tell the doctors and all that. They'll think I were well, but really it were I'd been having a few puffs of a joint [cannabis cigarette]." *(Participant 6)

Participant 13 described above said that medication was "bad", he continued:

"I've been on it for quite a while, [yawns] years and it doesn't seem to do anything for me because the amphetamine just counteracts it and over-powers it. It makes you look up like that sometimes [demonstrates eyes rolling]."

When asked what medication ought to do for him he said:

*"Give you something like amphetamine does for me, that's why I found the drug to cure me and to help me: amphetamine. Whereas this stuff they give me, depixol [antipsychotic depot], I don't know what it does to you, I don't feel any different"*. (Participant 13)

Seven were intending to abstain in the future and five of these said that they got benefit from their current medication. For instance, an adolescent white male, who had used cannabis since age 14, to join in with a delinquent group of peers, and had recently started using cocaine, agreed to take his antipsychotics short term and had ideas that using cannabis was part of returning to normality, but by the end of the interview decided he would quit for his 21^st ^birthday:

*"Because I've been using it for so long and um it's been, I've been through a lot, I've been through mental disease because of cannabis and I've been 'schiz', I don't know if its 'schiz', something like 'schiz'. But I've been through a lot, to give it up could be a standing point [good thing] for myself. Like saying, right I've been through a lot, this has put me through a lot this weed, put my family through a lot, I'm going to kick [stop using] it for good." *(Participant 10).

Conversely, ten intended to continue substance use in future and out of these ten, only three said that prescribed medication was helping them. Participants drew comparisons between medication and illicit substances as having in common the aim of altering mental state, the one difference being whether or not the substance was prescribed. In scrutinising prescribed medication in this way, most found it was less useful or had worse side effects than illicit substances. Rarely in this sample did medication ameliorate voices, but participants tended to complain instead about the effect of medication on their mood. There was rarely any expectation that medication would influence voices, but despite this some had complied with taking it, although there was general suspicion of 'treatment plans'.

Participant 17 described above explained how he had used stimulants to overcome the side effects of antipsychotic medication:

*"Just the shaking and the way I was in myself, introvert in myself, very light spoken. The reason I started taking a lot of crack was because when I didn't have a stimulant in me I couldn't be forceful, I couldn't put myself out, I couldn't put myself across to people, very 'like that' [whispering] because I didn't have the confidence it just dampened; when I was on depixol [antipsychotic depot medication] it just stopped me, it just zonked [sedated] me. It was like having a wall in front of you every morning know with having to get through that wall before I could get to living and it was, oh it was horrible. Really, really horrible". (*Participant 17).

Side effects were a common source of dissatisfaction with medication. Some complained that medication was given in hospital without proper explanation, which seemed to reduce the credibility of the message of abstinence of professionals as well as damaging trust. Participants often considered themselves to be knowledgeable about substances, such as awareness that similar perceptual abnormalities could occur with street drugs and medication, but felt that this was dismissed by professionals

*"... mean a nurse can't even explain that because she's never had drugs at all. She just knows that if you take that for a while you're going to get well. Side effects, I talked to a young girl "don't worry I said, have you ever taken drugs?" she said "no" I said, "this is what it's like when you do drugs this is how you feel" [snaps fingers] snapped out of it, straight away"; *(Participant 6)

Some accepted medical advice against using cannabis. Others had agreed that street drugs may be harmful but intended to continue using them. However, some saw professionals as not having valid knowledge about drugs and hence discredited their advice. First hand experience of substances and symptoms was often thought of as more valid than professional opinion. A white male in his late 30 s, who regularly used cannabis to manage his anxiety and feel more musical, had difficulty controlling the frequency of use because cannabis was "*psychologically addictive*". When the interviewer offered that she had read that cannabis was also physically addictive as evidenced by tolerance and withdrawals, he joked "*so it's best to smoke it every day*"(Participant 4).

### Reasons for abstinence

Our study aimed to elicit factors that maintain illicit drug use. However, 7 of the 17 participants reported a current intention to maintain abstinence. Reasons included disliking the effects and illegality of cannabis, financial benefits of abstaining, increasing age (five of the seven were age 35 years or over), physical health problems and negative impact on mental state. Four reported current family support and/or hope for relationships if they abstained and five reported hoped for or actual improved occupational/accommodation status.

Participant 17 explained that following a family bereavement, he had moved in with a relative, had a change of medication to one with fewer side effects and commenced voluntary work for a drugs service, thus he had been abstinent for the last 2 years:

*"Well I come from I look back at where I've been and what I've done who I was then. it was a combination of mental illness and the drugs, though it wasn't just the mental illness, it wasn't just the drugs, it was the combination of the two. it was the all the shit [bad] that lies with being a junkie [drug addict] like the stigma and how people look at you; kids coming up [saying] "smack head [heroin addict]" and throwing things at you. And you look like a tramp, you are a tramp basically, not everyone, but I was anyway, through the drugs, not eating. I've ate cat food. I've lived on cat food you know eating tins of cat food on bread, when I look at the person I was then, I'm not today. where I've come and that's what keeps me going, it's what keeps me off the drugs today, there's no way I'm going back there, I'm not going back to that because with me I know for me anyway at least with me, one's too many and a million's never enough, I can't just do one, it's all or nothing really"*. (Participant 17)

## Discussion

We have identified five interrelated factors that explain the maintenance of drug misuse in people with a diagnosis of schizophrenia. It was striking that the interviewees' decisions to use or abstain from substances were readily understandable when placed in the context of their life experiences and beliefs. The relative importance of each of our five factors varied between participants, but they best explained the substance misuse when taken together as headlines for the individual's stories.

### Relevance to the literature

Other qualitative studies which investigated drug use in people with psychotic disorders described as having a 'severe mental illness' are the COSMIC group study in inner London predominantly from Black Minority Ethnic groups [16], a larger American study [17] and a recent study of people in an early intervention service in the UK [15]. The COSMIC study interviewed fewer participants (14 participants), focussed primarily on drug use rather than experience of symptoms and included non specific psychosis and bipolar illness [16]. The USA study included people with primary diagnosis of anxiety/depression and people who did not use substances [17]. In the early intervention study, of the total sample of 19, several participants again appeared to have diagnoses other than schizophrenia [15].

As in earlier studies [16] the factors associated with onset of drug use in our study were consistent with those of the general population - exposure to drugs and influence of social networks [21]. Experience or knowledge of drugs give the individual 'social currency' [16] and a sense of belonging to a social network- a label of 'drug user' being preferable to that of mental illness [22]. However mental health settings may actually provide a venue for accessing substances and forming social networks with peers who use substances [9]. Our participants also used drugs to relax, achieve a pleasurable mental state and a sense of belonging [7,15,23] and our findings confirm a hierarchy of acceptability of substances [15], with cannabis as the most acceptable. However we also found that cannabis was perceived as a means of avoiding 'harder' drugs, because peer pressure could be assuaged and some intoxication achieved through its use. In addition we found that in some cases, pride in connoisseurship of substances (particularly cannabis) maintained substance use.

Our results partly confirm the suggestion that people who have schizophrenia may find it easier to develop social networks with drug users, who may be more tolerant than other social groups and more accepting of unusual personal attributes [24]. However, many of our participants reported hiding some symptoms from drug-using peers to avoid being labelled as ill.

A recent study [25] had shed doubt on the idea that good pre-morbid functioning in schizophrenia increased the risk of substance use [26]. Our findings concur with Lobban et al. [15] in that drug use commonly started with a group of peers but that all participants had some current difficulty with socialising and that drugs were used to alleviate this. This is consistent with studies showing that street drugs are used to enable positive social experiences and be accepted socially [16,27,28] and improvements in social functioning lead to decreased substance use [29].

A sub-group of people who have severe mental illness and who feel alienated from conventional services and society, use illicit substances, have unstable, impoverished social circumstances and see little chance of their ever gaining employment [30]. Loss, rejection and being exploited are experiences that can maintain or escalate drug use [31]. Reducing or stopping drug use can be motivated by a change in personal life goals [15], however, in our sample, there were examples of cycles of unattainable goals, disappointment and relapsing to drug use to cope with such feelings. It was difficult to establish why they had settled upon such goals, as any challenge to these seemed to threaten rapport during the interviews. However it seemed that they did not believe they deserved such impoverished lives and felt entitled to better, but they were outside of and thus unable to learn from any culture of working steadily towards realistic goals. In addition, we found drugs were sometimes used to reminisce about happier times. Thus our study highlights the long term challenge of maintaining any reduction in drug use.

Previous studies have found good evidence that clients with schizophrenia use substances to self medicate for tension, low mood, anxiety and negative symptoms [6,10,15,16,27] as did we. However studies of whether or not self-medication occurs for positive symptoms have revealed mixed results [10,16]. This may reflect differences in methodology. We found that some patients were aware of adverse effects of drugs on positive symptoms [16,22,20] but that substance use did not exacerbate positive symptoms in all individuals [1,15,20,32]. However we found that a particular client may regard some symptoms as being illness (such as depressed mood) and others as being real (such as voices) and they may be unwilling to give up substances that increase certain symptoms because of a reliance upon anxiolytic effects of substances, for instance to cope with hearing voices. Hence participants could see themselves as using drugs to cope with the stress of 'real' persecution. Unlike in other qualitative studies we also did identify cases of use of drugs with the aim of reducing hearing voices, (note that this did not necessarily involve the participant viewing the voices as being due to any kind of illness).

Participants told us that street drugs had to be used at optimal doses to achieve beneficial effects on mood and to avoid 'paranoia'. Our results concurred with previous qualitative research [15] that personal experience of substance use effects and the temporal sequence of events were given more credibility than professional opinion about influence of drugs on a person's mental state. In our study, participants also said that there was an optimal dose but admitted that it was difficult to not escalate the dose of street drugs, to their detriment. Therefore, cutting down may sometimes be a more realistic goal than abstinence. Difficulty was encountered in adhering to the optimal dose of the chosen substance despite participants often perceiving themselves to be experts in that drug, the drugs themselves seemed to be seen as inherently challenging and slips were a part of an explorer's experience, as if they were mountain climbers. Tactful discussion of such proneness to slips could be put to therapeutic use with patients.

We also found that some participants were using stimulants to enable them to engage in activities, this may be a vicious circle as cocaine has been shown to increase extra-pyramidal side effects of antipsychotics [33] and some patients did cite extra-pyramidal side effects as a reason for drug use [16]. Patients may also view illicit drugs as more efficacious against subjective distress than prescribed drugs [34-36]. We found that professionals may be seen as hypocritical and controlling for discouraging street drug use whilst insisting on medication compliance. The implication of this finding is that a non-collaborative approach to pharmacotherapy may result in street drug-taking that is in part a rebellion against the clinician.

Some participants suggested that they used cannabis in the hope of reducing their voices as they had intermittently had this effect in the past. Intriguingly, cannabidiol reduces the effect of THC, the main hallucinogenic constituent of cannabis; cannabidiol has antipsychotic properties [37], but the THC content is increased and the cannabidiol content is reduced in currently available street cannabis compared to ten years ago [7]. Could it be that patients previously found benefit from higher cannabidiol content cannabis and are still using cannabis on that basis?

Some participants reported street drugs to focus their attention more on persecutory voices in the hope of outwitting their perceived persecutors. The phenomenon of using drugs to increase voices had been noted in a previous study [18] and our study has confirmed and elaborated this finding.

Finally, our results also offer some support for the hypothesis that a common factor may simultaneously increase the risk of drug use and of schizophrenia [6]. Many participants spoke of childhood adversity, including early traumatic experiences, family dysfunction, deprivation and poor educational attainment, all of which have been linked with both schizophrenia and substance use disorders [6].

### Strengths and limitations

We have presented our findings to a group of service users who validated the themes that we identified. To our knowledge, this study is unique in using qualitative analysis of interviews to investigate reasons for on-going drugs use in people who all have a clinical diagnosis of schizophrenia. Previous studies with mixed populations have left doubts as to whether the themes identified applied to people with schizophrenia. We assert that we have clarified and expanded our knowledge of reasons for drug use in people with schizophrenia and therefore demonstrated the usefulness of qualitative research in this subject area.

We accept that the sample size in this study was small, but saturation of themes was achieved in the analysis. Another limitation must however be the low numbers of ethnic minority and female participants that we succeeded in recruiting. While it is not suggested that the specific results are generalisable to all populations, we have attempted to delineate the range of views that people with a diagnosis of schizophrenia who use drugs may commonly hold and the explanations they might have for these views.

Our study did not specifically assess for research diagnostic criteria for a diagnosis of schizophrenia, clinical or research criteria of drug dependence or for presence of co-morbid personality disorder.

### Reflexivity

The first author carried out all of the interviews and may have become progressively more attuned to the participants' stories as the data collection went along. The first author informed all participants of her job title but then encouraged use of her first name and adopted a friendly and 'curious' stance. This was to avoid non-disclosure of data due to thinking that she would disapprove. However, when participants seemed to be strongly talking themselves into increased drug use, for ethical reasons, the interviewer tended to encourage balancing this against 'less good things' they could identify about drug use. Both authors are psychiatrists, so in analysing the data and so we would tend to see medical symptom clusters, such as anxiety and depression, amongst the reasons given for street drug use.

## Conclusions

The use of qualitative methods in dual diagnosis research is supported. This study has identified novel factors that maintain drug use in schizophrenia, as well as usefully confirming some of the findings of recent qualitative research with people with 'psychosis'. Familiarity with our five themes generated from this study could improve the mental health professional's clinical assessment of dual diagnosis patients, in terms of gathering more pertinent information and being sensitive to the client's perspective in collaborating formulating a management plan. Further studies are warranted to evaluate standardised methods of assessing patients for the presence and relative importance of the five reasons for continuing drug use identified in our study, with a view to improving outcomes for this population.

## Competing interests

The authors declare that they have no competing interests.

## Authors' contributions

CJA designed and conceived of the study and carried out the interviews. Both authors read all transcripts and carried out analysis of the data. We can confirm that we both had full access to the data in this study and CJA takes full responsibility for the integrity and accuracy of the data analysis. CJA drafted and both authors revised and approved the final manuscript.

## Authors' information

LG, MB ChB MSc PhD FRCPsych, is Professor of Primary Care Psychiatry, jointly appointed in both psychiatry and primary care, at University of Manchester and works as a Consultant Psychiatrist in the Primary Care Mental Health Service in Salford.. CJA, MBBS BSc MRCPsych MSc, completed training in General Psychiatry and Psychotherapy and is currently employed as a locum consultant psychiatrist in Crisis Resolution/Home Treatment, at Pennine Care NHS Foundation Trust

## Pre-publication history

The pre-publication history for this paper can be accessed here:

http://www.biomedcentral.com/1471-244X/10/94/prepub
